# Plasma metabolomic analysis of human hepatocellular carcinoma before and after transcatheter arterial chemoembolization

**DOI:** 10.7150/ijms.89141

**Published:** 2024-01-01

**Authors:** Jing Fan, Min Xu, Sizhu Lu, Mengxuan Shan, Ke Liu, Wanping Yan, Wei Ye

**Affiliations:** 1 Clinical Research Center, The Second Hospital of Nanjing, Affiliated to Nanjing University of Chinese Medicine, Zhong Fu Road, Gulou District, Nanjing, Jiangsu 210003, China.; 2 Department of infectious disease and liver disease, The Second Hospital of Nanjing, Affiliated to Nanjing University of Chinese Medicine, Zhong Fu Road, Gulou District, Nanjing, Jiangsu 210003, China.

**Keywords:** hepatocellular carcinoma, TACE, metabolomic, plasma

## Abstract

**Introduction:** Hepatocellular carcinoma (HCC) is the fourth most prevalent cancer in China. Transcatheter arterial chemoembolization (TACE) is a common interventional therapy for HCC. In this study, we aimed to explore specific metabolites that can accurately predict prognosis after TACE in patients with HCC.

**Methods:** Patients with HCC and healthy volunteers (n = 20 each) were recruited to our study; plasma samples were collected from patients before and after TACE and from healthy volunteers. Plasma samples were subjected to untargeted ultra-high performance liquid chromatography-high resolution mass spectrometry metabolomics analysis, to identify metabolites significantly associated with the prognosis of patients with HCC after TACE.

**Results:** Orthogonal filtered partial least squares discriminant analysis confirmed significant separation of the pre-TACE, post-TACE, and healthy groups, and 34 differential metabolites were identified between the pre-TACE and post-TACE groups. KEGG analysis revealed that phenylalanine, tyrosine, and tryptophan biosynthesis pathways and the phenylalanine metabolism pathway were potentially altered in HCC genesis and during TACE. Phenylalanine and tyrosine are involved in both pathways and were increased in the pre-TACE group relative to controls, with phenylalanine further increased in the post-TACE group. Receiver operating characteristic (ROC) curve analysis indicated that PC 36:4|PC 18:2_18:2 (area under the ROC curve (AUC) = 0.798) is a potential marker for assessment of prognosis in patients with HCC after TACE. Moreover, ROC curve analysis indicated that palmitoylcarnitine (AUC = 1) is a marker with potential value for HCC diagnosis.

**Conclusions:** Limited studies had been conducted on the detection of metabolites in the plasma of HCC patients before and after TACE. PC 36:4|PC 18:2_18:2 is a potential marker for evaluation of the therapeutic effects of TACE. This finding may be beneficial for the treatment of patients with HCC after TACE.

## 1. Introduction

Hepatocellular carcinoma (HCC) is among the most common gastrointestinal malignancies, with the seventh highest incidence worldwide and ranking third for mortality^1^. HCC has a high rate of recurrence, mortality, and metastasis in China^2^. Common treatments for HCC include hepatectomy, liver transplantation, transcatheter arterial chemoembolization (TACE), ablative therapy, radiotherapy, and systematic antitumor therapy, and among others^3^. Since HCC tumors are often detected at an advanced stage or when they are not suitable for surgical excision, interventional radiology is often used for patients with intermediate or advanced HCC. TACE, as a type of interventional surgery, is the first line treatment for intermediate stage HCC, according to the Barcelona Clinic Liver Cancer system and the Chinese University Prognostic Index^4^. In TACE, the hepatic artery is embolized to cut off the blood supply to the HCC tumor, leading to ischemia and hypoxia, which inhibit tumor growth and can even induce tumor necrosis. Moreover, embolization drugs combined with chemotherapy during TACE can further reduce the tumor burden.

Metabolomics is an emerging technology that allows quantitative analysis of all the metabolites in an organism at a given moment, to analyze relative relationships between metabolites and physiological and pathological changes. Metabolite analysis of biological fluid can directly and accurately reflect the pathophysiological state of the subject. By comparing metabolites at different stages, changes can be determined, and the biochemical characteristics of each stage can be elucidated. Since its emergence, metabolomics has been widely used to detect alterations metabolite levels, which can indicate the presence of diseases, such as melanoma, oral squamous cell carcinoma, and lung cancer^5^,^6^,^7^. This technique is also widely used in HCC. Various differential metabolites, including phenylalanine-tryptophan, urea, and retinol, are considered potential biomarkers for HCC^8^,^9^,^10^. These metabolites and their related enzymes have important roles in HCC diagnosis and prognosis and have provided a theoretical basis and therapeutic indicators for future diagnosis and treatment^8^,^11^,^12^,^13^. For example, Wang *et al* developed a scoring system that combines phenylalanine and choline with the presence of satellite nodes to assess overall survival of patients with HCC^14^. Another study suggested that there was a positive correlation between purine, pyrimidine, and amino acid metabolism and HCC metastasis^7^. Clinical response to treatment and recurrence or metastasis of HCC may also be traced by metabolite detection in body fluid samples; however, there have been few studies on the metabolomics of patients with HCC before and after TACE. We hypothesized that TACE would change tumor metabolic pathways or affect tumor environmental metabolism, thus playing a role in tumor therapy.

In this study, we employed untargeted metabolomics to investigate the biochemical changes in plasma of patients with HCC before and after TACE. We also compared the plasma metabolites of healthy individuals and patients with HCC, to identify abnormal metabolites associated with HCC development. Further, we analyzed the alterations in metabolites in patients with HCC before and after TACE, to understand the mechanism underlying TACE therapy. Additionally, we used differential metabolite and pathway analysis to determine pathways potentially affected by TACE. Finally, we conducted receiver operating characteristic (ROC) curve analysis, to identify markers with potential for application in evaluating the therapeutic effects of TACE.

## 2. Methods and Materials

### 2.1. Study cohort

Plasma samples from 20 patients with HCC were collected before and 7 days after TACE. In addition to TACE, all patients with HCC received general liver protection treatment. Plasma samples from 20 healthy volunteers were collected as the control group. All patients with HCC and healthy volunteers were recruited from the Second Hospital of Nanjing, Nanjing University of Chinese Medicine, from September 2020 to September 2021. Patients with HCC ranged in age from 45 to 75 years, and there were no significant differences in age or sex between the patient and control groups (Table 1). Diagnosis of HCC was based on alpha-fetoprotein level, computed tomography/magnetic resonance imaging, and histopathological changes. Individuals with severe cardiopulmonary insufficiency or coagulopathy or cachexia were excluded. Further, enrolled healthy volunteers had no current or past history of liver disease, infectious diseases, or metabolic disorders, and had not used medication that may affect liver metabolism. Clinical information was collected from the enrolled subjects. Tumor response was assessed by radiographic findings at 4-6 weeks postoperative, according to response evaluation criteria in solid tumours. This study was approved by the medical ethical committee of the Second Hospital of Nanjing, Nanjing University of Chinese Medicine, and written informed consent was received from all subjects.

### 2.2. Plasma sample preparation and quality control (QC)

Plasma samples (n = 60) were analyzed using an ultra-high performance liquid chromatography-high resolution mass spectrometry (UHPLC-HRMS/MS)-based untargeted metabolomics platform. All enrolled subjects followed overnight fasting. Methanol (200 μL) was added to 50 μL plasma samples, and vortexed to mix evenly. Then, samples were incubated at -40 ℃ for 30 min and 4 ℃ for 10 min, before centrifugation (15000 *g*, 4℃, 15 min). Supernatants were evaporated to dryness, and samples then reconstituted in 50 μL 10% methanol. A QC sample was obtained by isometric pooling of all prepared samples. Internal standards were valine-^13^C_5_-^15^N, leucine-^13^C_6_, phenylalanine-d_5_, 3-chloro-D-phenylalanine, octanoic-d_15_ acid, decanoic-d_19_ acid, octadecanoic-d_35_ acid, tetradecanoic-d_27_ acid, hexadecanoyl-L-carnitine-d_3_, and decanoyl-L-carnitine-d_3_.

### 2.3. UHPLC-HRMS/MS platform

Chromatographic separation of all samples was performed using a ThermoFisher Ultimate 3000 UHPLC system with a Waters ACQUITY UPLC BEH Amide column (2.1 × 100 mm, 1.7 μm). Mobile phases were solvent A (water containing 15 mM ammonium acetate (pH = 9)) and solvent B (90% acetonitrile containing 10 mM ammonium acetate). Linear gradient elution was performed as follows: 0 min, 90% B; 4 min, 85% B; 11-18 min, 75% B; 18.1-20 min, 90% B. Flow rate was 0.25 mL/min. Eluents were analyzed on a ThermoFisher Q Exactive™ Hybrid Quadrupole-Orbitrap™ Mass Spectrometry in heated electrospray ionization positive and negative modes, separately. The main parameters were set as follows: (1) spray voltage, 3500 V; (2) capillary and probe heater temperature, both 350°C; (3) sheath gas flow rate, aux gas flow rate, and S-Lens RF level, 40 (Arb), 10 (Arb), and 50 (Arb), respectively; (4) full scan was operated at high-resolution (35000 FWHM, m/z = 200), range 70-1050 m/z, with AGC target, 3 × 10^6^. Data-dependent acquisition was used to acquire fragment ion information from up to 8 precursors in each scan. Parameters were as follows: HCD energy, 15, 30, and 45 eV; mass resolution, 17500 FWHM; and AGC threshold, 2 × 10^5^.

### 2.4. Data processing and identification of UHPLC-HRMS/MS data

ProteoWizard software was used to transform the UHPLC-HRMS/MS data to mzXML format. The XCMS and CAMERA packages were employed in the R software platform, to further process the data, and final data were compiled as a peak table file, containing observations (sample name), variables (retention times (RT)_mass to charge ratio (m/z)), and peak areas. Peak area data were normalized to internal standards before performing univariate or multivariate statistical analysis. All results with relative standard deviation ≥ 30% within the repeated injections of QC samples were removed.

MS-DIAL software was used to identify metabolites. Accurate mass to charge ratio (m/z) of parent ions, mass spectra of fragment ions, and RT were matched against mzCloud, MoNA, and HMDB databases. Tolerances of accurate mass to charge ratio (m/z) for parent ion and mass spectra of fragment ions to theoretical mass to charge ratio were limited to 0.005 Da and 0.05 Da, respectively. The matching similarity threshold for mass spectra of fragment ions was set at 70%. For the in-house database, RT tolerance was set to 0.5 min. For public or commercial databases, RT tolerance was set to infinity.

### 2.5. Statistical analysis

Data were normalized by PAR scaling before undergoing multivariate statistical analysis. Principal component analysis (PCA) and orthogonal filtered partial least squares discriminant analysis (OPLS-DA) were performed using normalized data. Model quality is described using R^2^X or R^2^Y and Q^2^ values. Student's *t* test or paired *t* test was used to evaluate the significance of differences between two groups and *p* values exported. Screening criteria defining differential metabolites between two groups were: fold change > 1.2 and *p* < 0.05. Pathway analysis was performed using the MetaboAnalyst 5.0 (http://www.metaboanalyst.ca/) online tool. ROC curves were calculated and plotted using Medcalc software. Relative quantification of differential metabolites was plotted using GraphPad Prism 9.0.

## 3. Results

### 3.1. Clinical characteristics of study participants

A total of 20 patients with HCC (14 males and 6 females; mean age, 61 years) were recruited to this study and 20 healthy volunteers (13 males and 7 females; mean age, 57 years) were recruited as the control group. There was no significant difference in sex or age between patients with HCC and healthy volunteers (Table 1). No differences in biological or histological parameters were detected between patients with HCC pre- and post-TACE (Table 1).

### 3.2. UHPLC-HRMS/MS-based metabolic profiling

**Figure 1:**
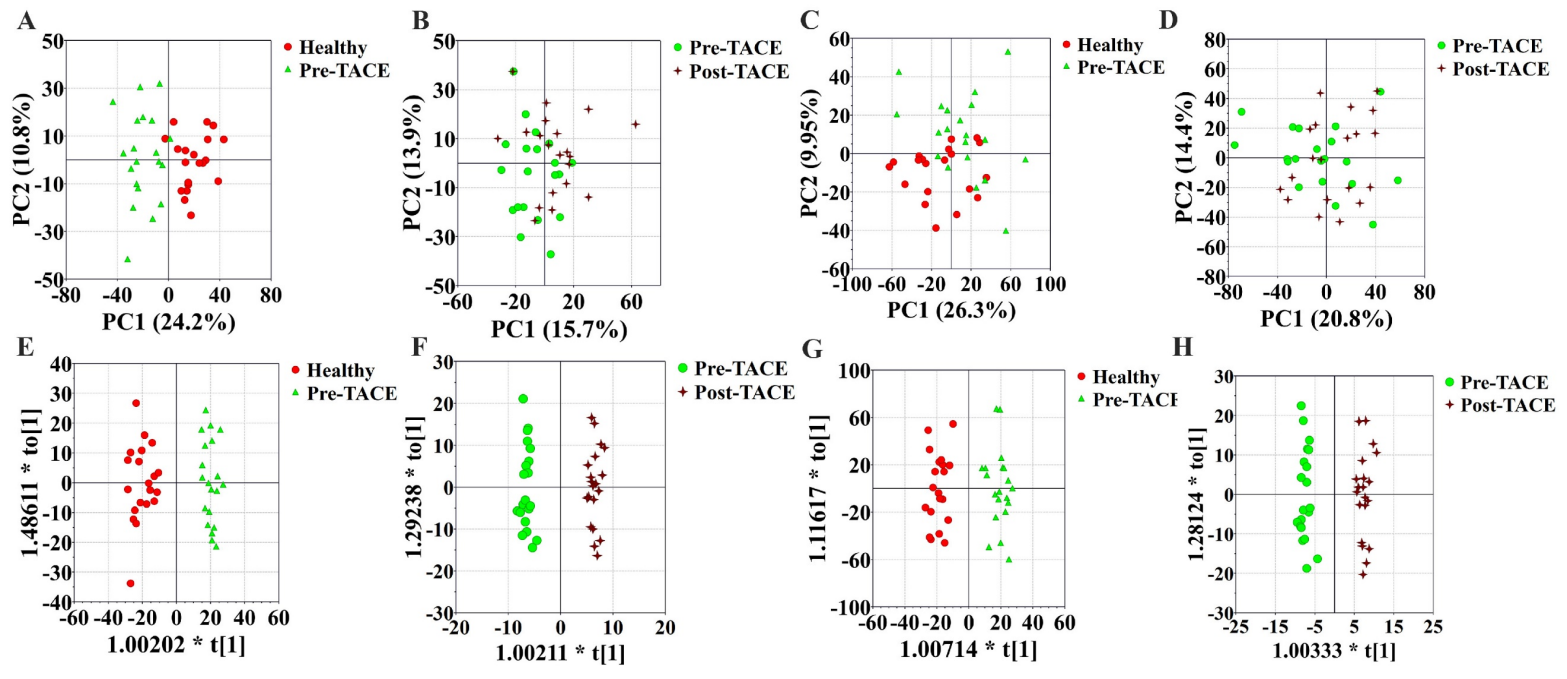
** Metabolic profiling.** (A-B) PCA analysis, positive ion mode. 5 principal components for the comparison in (A) and 8 principal components for the comparison in (B); (C-D) PCA analysis, negative ion mode. 5 principal components for the comparison in (C) and 6 principal components for the comparison in (D); (E-F) OPLS-DA analysis, positive ion mode.; (G-H) OPLS-DA analysis, negative ion mode. There was one predictive component and one orthogonal component used for the comparison in (E) and (G), and one predictive component and three orthogonal components used for the comparison in (F) and (H).

To discover differences in metabolic patterns in patients with HCC before and after TACE, we used UHPLC-HRMS/MS to compare metabolites among the healthy, pre-TACE, and post-TACE groups. Normalized data were preprocessed by par scaling and mean centering. PCA plots for these three groups and QC data are presented in Supplemental Figure 1. PCA score plots in positive and negative ion modes suggested distinct clustering of samples between the healthy and pre-TACE groups (R^2^X = 0.532 in positive ion mode, R^2^X = 0.521 in negative ion mode), but not between the pre- and post-TACE groups (R^2^X = 0.651 in positive ion mode, R^2^X = 0.546 in negative ion mode) (Fig. 1A-D). OPLS-DA was further used to analyze metabolite data from the different groups. The resulting OPLS-DA model could distinguish samples between the healthy and pre-TACE groups (R^2^X = 0.361, R^2^Y = 0.947, Q^2^ = 0.771 in positive ion mode, R^2^X = 0.414, R^2^Y = 0.938, Q^2^ = 0.789 in negative ion mode) and between the pre- and post-TACE groups (R^2^X = 0.349, R^2^Y = 0.983, Q^2^ = 0.568 in positive ion mode, R^2^X = 0.356, R^2^Y = 0.975, Q^2^ = 0.514 in negative ion mode), (Fig. 1E-H). Moreover, the results of permutation tests for OPLS-DA model shown in Supplemental Fig. 2 and all the p value of CV-ANOVA for OPLS-DA was <0.05. So, these results indicated that there were significant differences in metabolites among the groups.

### 3.3. Discovery of metabolic profiles of different groups

**Figure 2:**
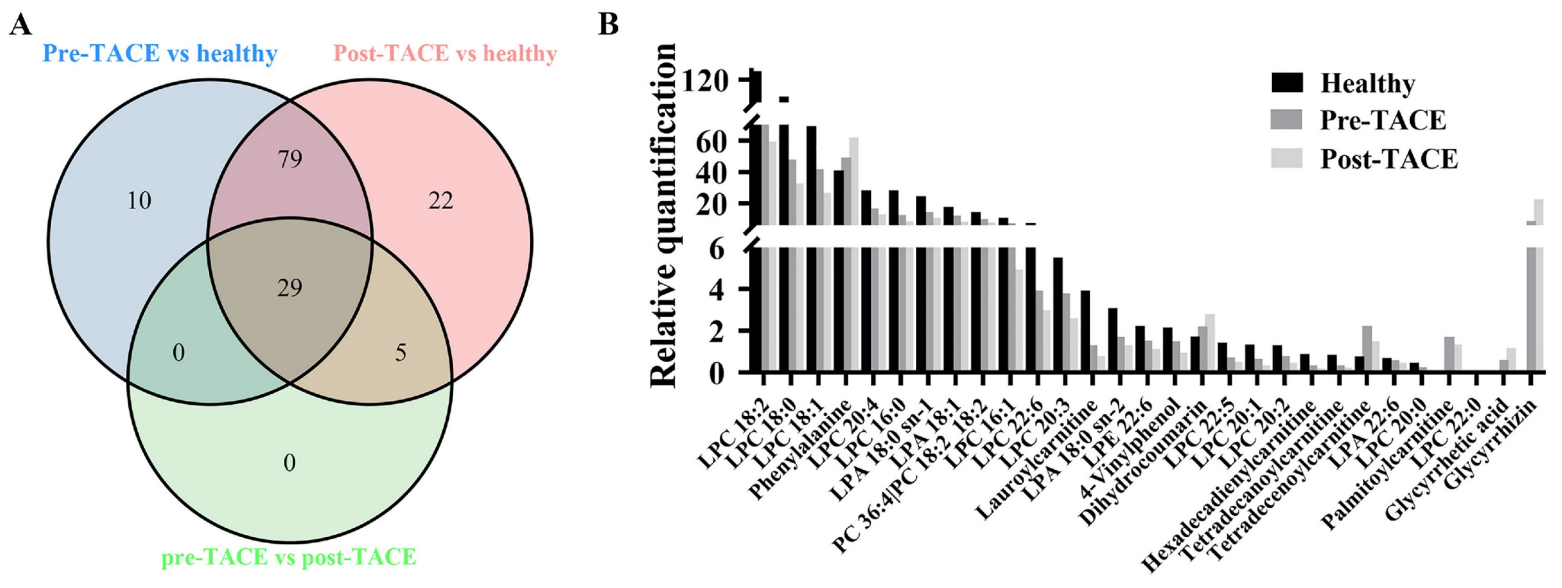
** Differential metabolites among the healthy, pre-TACE, and post-TACE groups.** (A) Venn diagram of differential metabolites among the healthy, pre-TACE, and post-TACE groups. (B) Relative quantification of 29 differential metabolites in the three groups.

**Figure 3:**
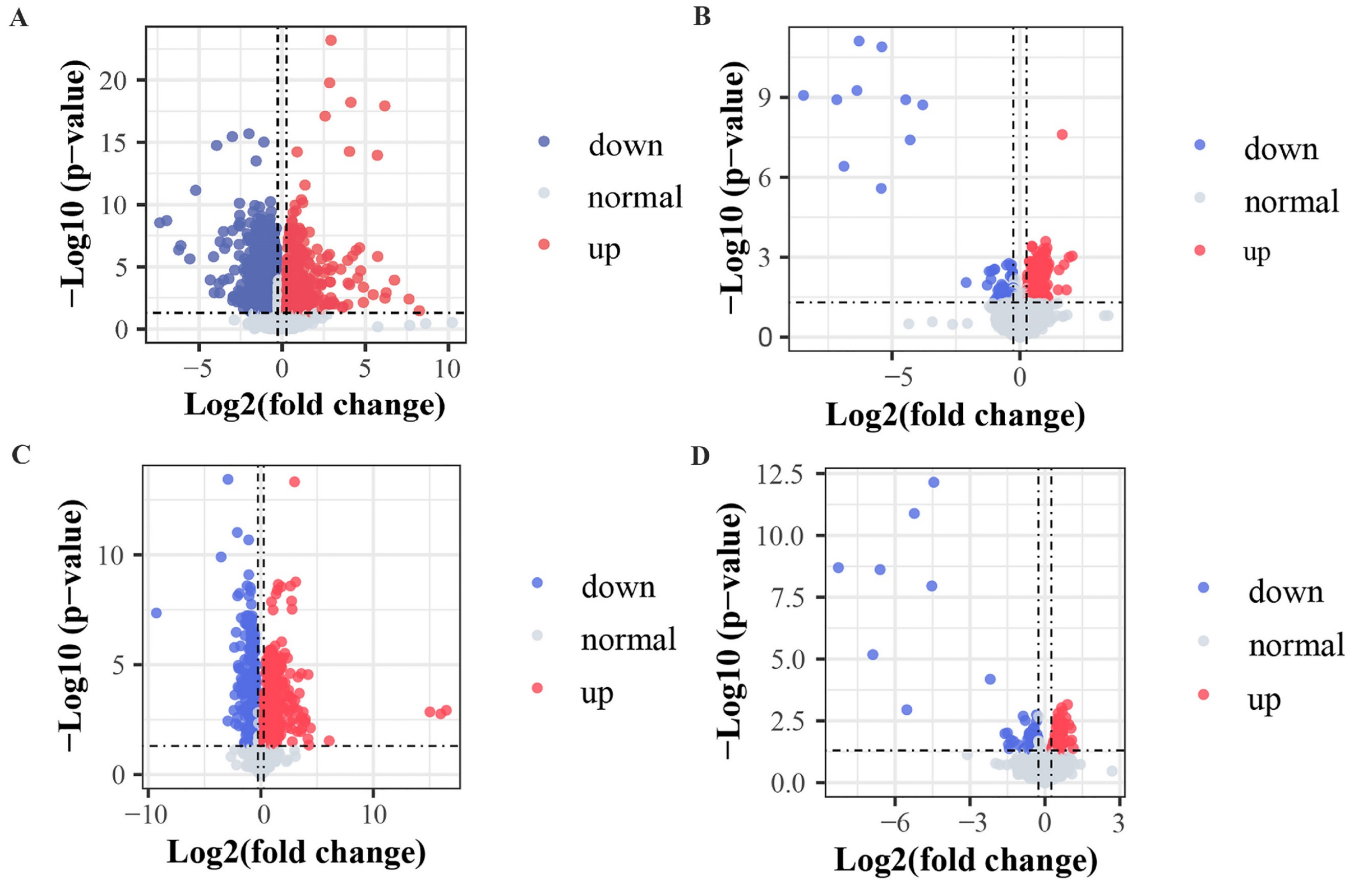
** Volcano plots comparing metabolites among different groups.** (A and C) Pre-TACE group compared with the healthy group. (B and D) Post-TACE group compared with the pre-TACE group. (A-B) Positive ion mode. (C-D) Negative ion mode. Threshold parameters: fold change > 1.2, *p* < 0.05.

The criteria for identification of differential metabolites among the analyzed groups were set as fold change > 1.2 and *p* < 0.05. The obtained data indicated that 29 metabolites exhibited clear variation in levels among the healthy, pre-TACE, and post-TACE groups (Fig. 2). Further analysis of the pre-TACE group versus healthy group revealed that 118 metabolites differed significantly, of which 96 were decreased and 26 were increased (Fig. 3A and C) (Supplemental Fig. 3). Differences in metabolites before and after TACE in patients with HCC have not been reported. These metabolites may be useful to evaluate the therapeutic effect of TACE. To explore the metabolic changes during TACE, we compared metabolites between the pre- and post-TACE groups and found that 34 metabolites differed significantly, of which 4 were decreased, and 30 were increased (Fig. 3B and D) (Supplemental Fig. 4).

### 3.4. Metabolic Pathway Analysis

**Figure 4:**
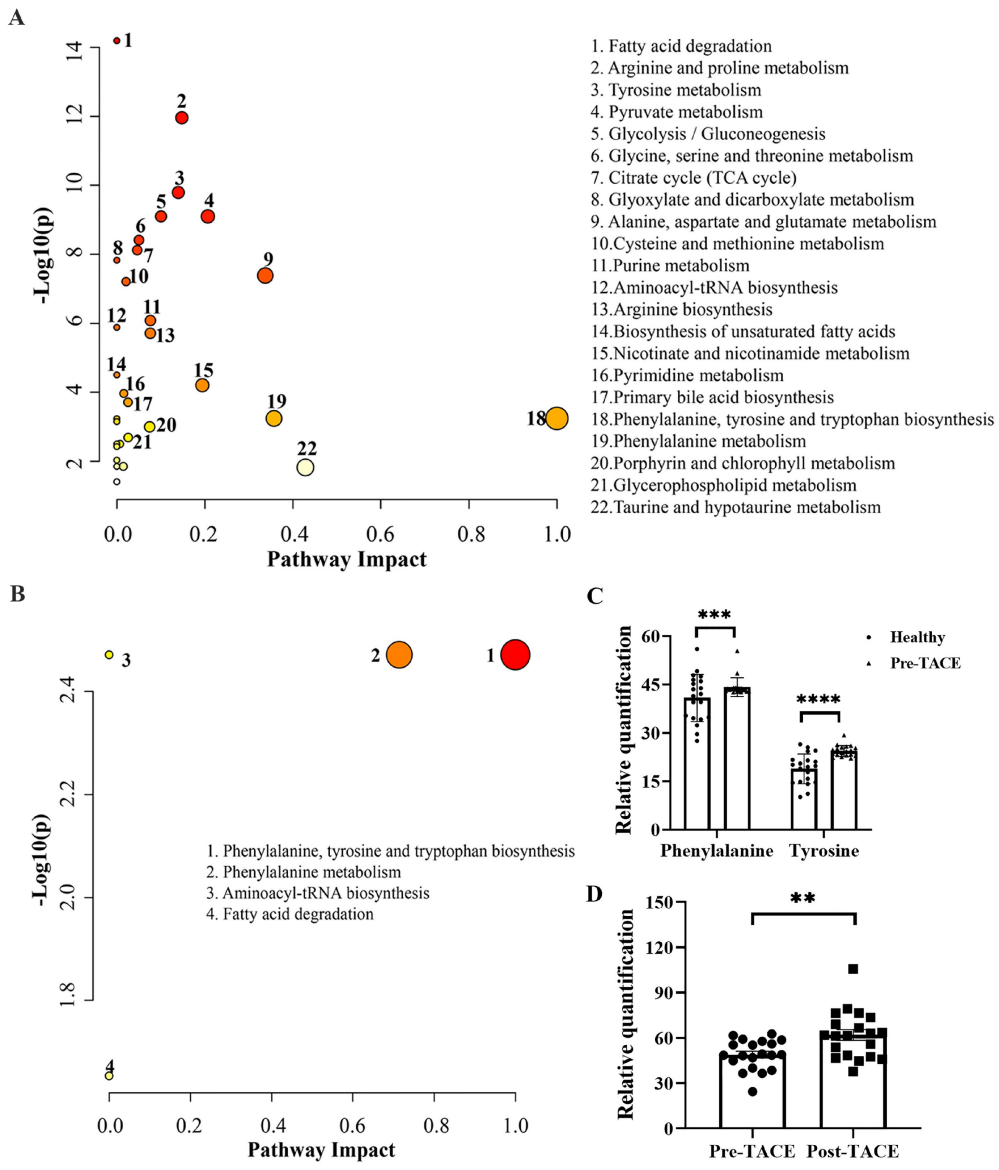
** Pathway enrichment analysis of differential metabolites between different groups.** Pathways enriched for differential metabolites (A) between the healthy and pre-TACE groups and (B) between the pre- and post-TACE groups. Abscissa values were based on pathway topology analysis, and indicate the importance of the pathway to the occurrence of the difference. Larger circles indicate higher importance. Ordinate values indicate the correlation between metabolic pathways and groups. Deeper red color indicates a stronger correlation. (C) Histogram showing the relative abundance of phenylalanine and tyrosine in the healthy and pre-TACE groups. Statistical analysis was performed using the student's *t* test. (D) Histogram showing the relative abundance of phenylalanine between the pre- and post-TACE groups. The paired *t* test was used for comparisons. **p < 0.01, ***p < 0.001, ****p < 0.0001.

To investigate potential pathways that may be significantly altered during HCC genesis and TACE treatment, MetaboAnalyst version 5.0 was used to identity pathways enriched for differential metabolites. KEGG analysis between the healthy and pre-TACE groups showed that 22 pathways were significantly enriched. By combining -LOG(p) and impact values, we concluded that phenylalanine, tyrosine, and tryptophan biosynthesis (-LOG(p) = 3.24, impact = 1) and phenylalanine metabolism (-LOG(p) = 3.24, impact = 0.36) pathways may been involved in HCC genesis (Fig. 4A). Both pathways involved phenylalanine and tyrosine, which were increased in the pre-TACE group relative to the healthy group (Fig. 4C). Further, KEGG analysis of the pre- and post-TACE groups showed that four metabolic pathways were significantly enriched (Fig. 4B). According to -LOG(p) and impact values, the phenylalanine, tyrosine, and tryptophan biosynthesis and phenylalanine metabolism pathways may be altered during TACE (Table 2). Phenylalanine was involved in both pathways and was increased in the post-TACE group relative to the pre-TACE group (Fig. 4D). Given that many of the differential metabolites between the pre-TACE and post-TACE groups were lipid substances, we employed BioPan for a lipid pathway enrichment analysis. The results indicated that PC36:4|PC18:2_18:2 plays a significant role (Supplemental Fig. 5).

### 3.5. Potential biomarkers for HCC diagnosis and evaluation of TACE efficacy

**Figure 5:**
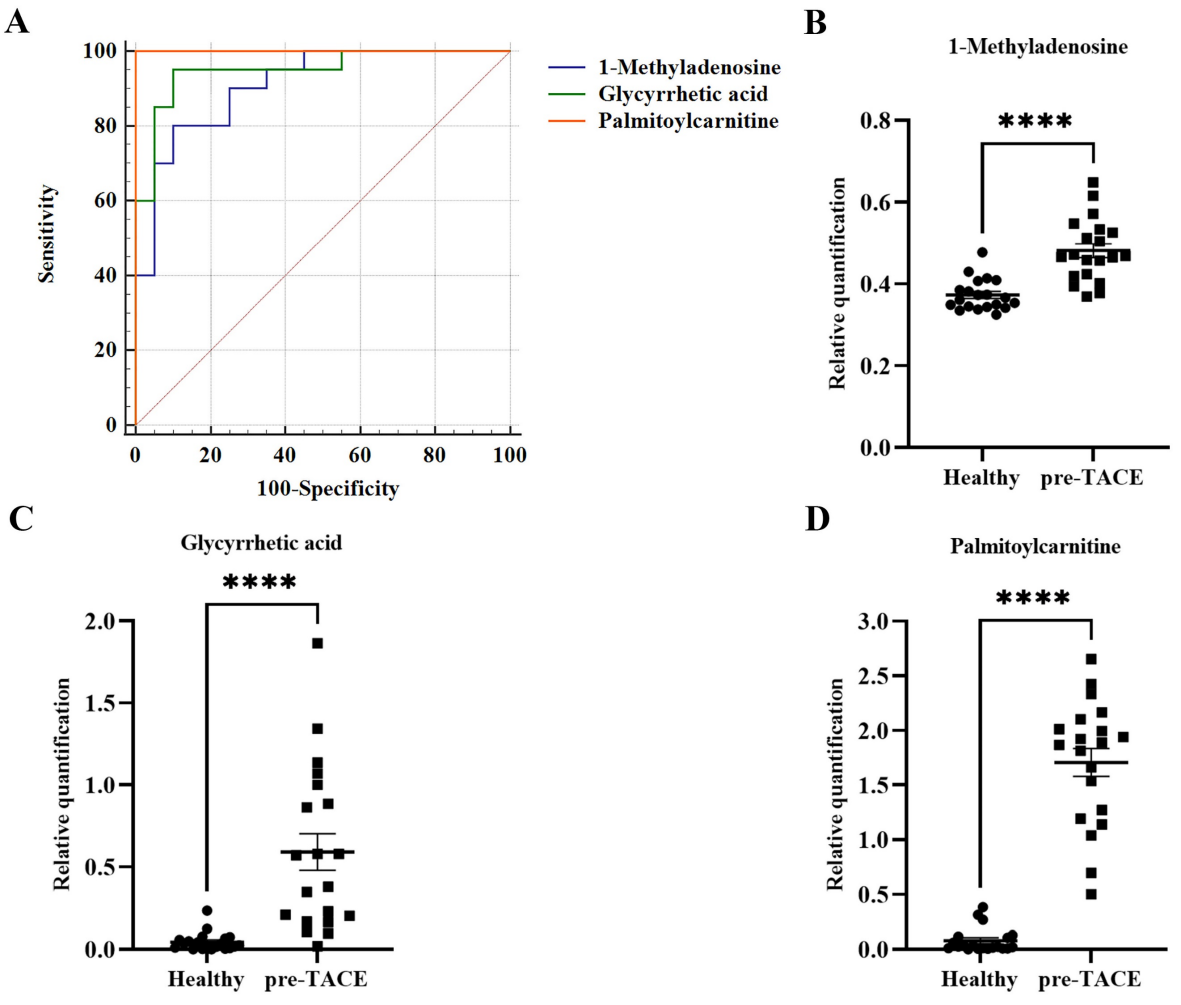
** Potential biomarkers for HCC diagnosis.** (A) ROC curves for differential metabolites between the healthy and pre-TACE groups. AUC_1-methyladenosine_ = 0.91; AUC_glycyrrhetic acid_ = 0.95; AUC_palmitoylcarnitine_ = 1.00. (B-D) Histograms showing the relative abundance of three differential metabolites between the healthy and pre-TACE groups. Statistical analysis was performed using the student's *t* test. ****p < 0.0001.

To discover potential biomarkers for diagnosis of HCC, ROC curves were plotted for the 118 differential metabolites between the healthy and pre-TACE groups, among which, three metabolites (1-methyladenosine, glycyrrhetic acid, and palmitoylcarnitine) had AUC values > 0.9 (*p* < 0.05) (Fig. 5A). Notably, all three of these metabolites were increased in the pre-TACE group compared with the healthy group (Fig. 5B-C). Together with the results of sensitivity and specificity analysis, our data led us to speculate that palmitoylcarnitine (AUC = 1) has potential as an auxiliary biomarker for HCC diagnosis; the cut-off value for palmitoylcarnitine was > 0.38.

**Figure 6:**
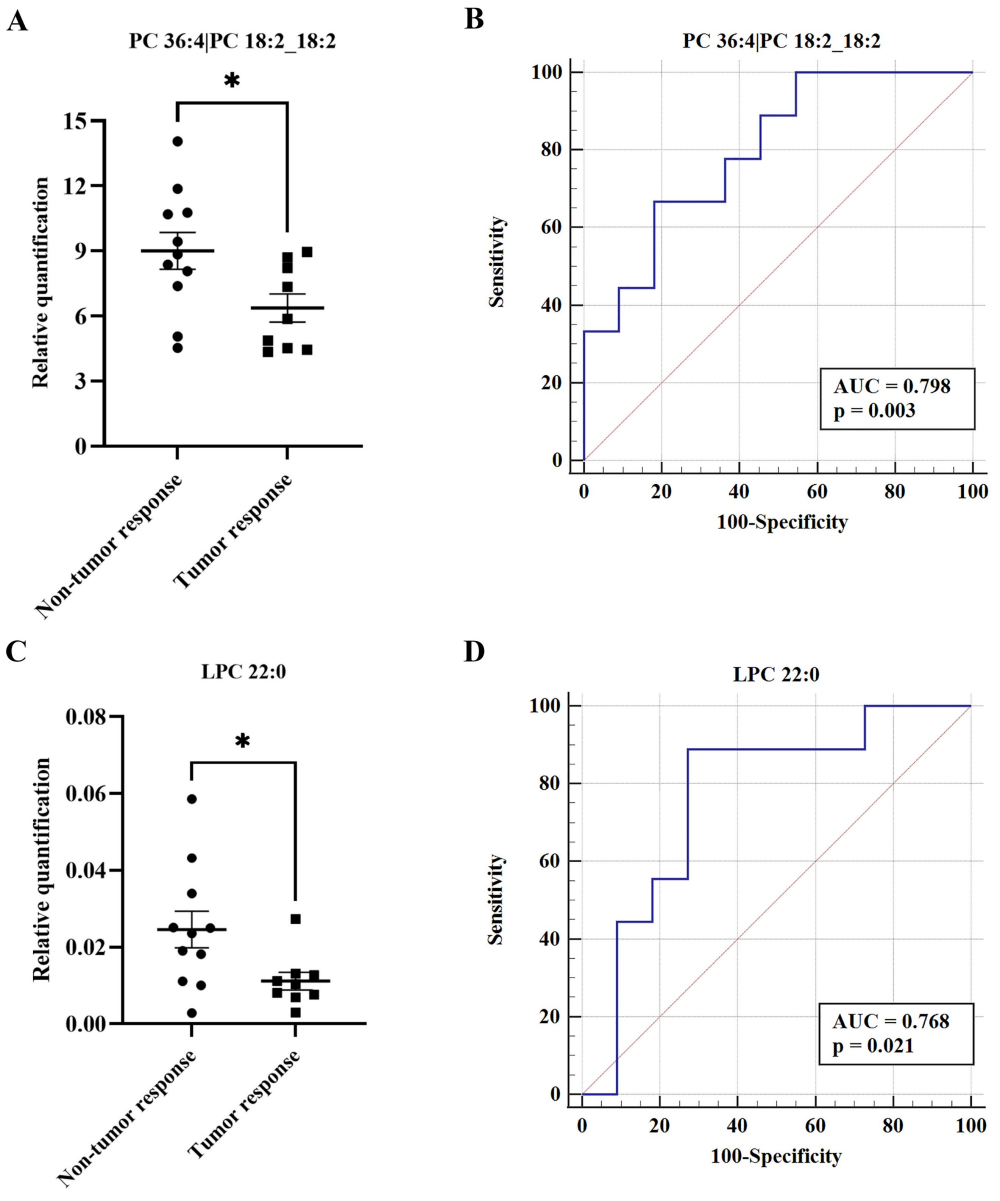
** Potential biomarkers to evaluate the therapeutic effect of TACE.** (A and C) Histograms showing the relative abundance of PC 36:4|PC 18:2_18:2 and LPC 22:0 between the non-tumor response and tumor response groups, respectively. The Student's *t* test was used for comparisons; *p < 0.05. (B and D) ROC curves for PC 36:4|PC 18:2_18:2 and LPC 22:0 between the non-tumor response and tumor response groups, respectively. ROC curves and AUC values were generated using Medcalc software.

To date, there are no effective indicators for evaluation of the therapeutic effects of TACE. Therefore, we divided the post-TACE group into non-tumor response (n = 11) and tumor response (n = 9) groups. Of 34 differential metabolites between the pre- and post-TACE groups, two metabolites (PC 36:4|PC 18:2_18:2 and LPC 22:0) differed significantly between the non-tumor response and tumor response groups (Fig. 6A and C). Furthermore, ROC curve analysis yielded AUC > 0.75 and *p* < 0.05 for these two metabolites (Fig. 6B and D). Given the low relative quantities of LPC 22:0 detected and the results of comprehensive sensitivity and specificity analysis, we favor PC 36:4|PC 18:2_18:2 as a metabolite potentially useful for evaluation of the therapeutic effects of TACE (Table 3).

## 4. Discussion

HCC is the fourth most common malignant tumor in China, with a high rate of metastasis and mortality^15^. TACE is a non-invasive interventional radiology treatment that limits the blood supply to tumors while delivering chemotherapy drugs^16^. Metabolic disorders occur during tumor progression, and metabolomics is a high throughput approach that measures metabolite concentrations in different pathological or physiological states to define the metabolic status of biological systems associated with diseases. In this study, untargeted UHPLC-HRMS/MS metabolomics analysis was performed on serum samples from 20 patients with HCC and 20 healthy volunteers to detect metabolic differences and predict patient prognosis after TACE.

OPLS-DA model analysis revealed clear distinctions among the groups. We identified 118 differential metabolites between the healthy and pre-TACE groups and 34 between the pre- and post-TACE groups. KEGG analysis revealed that the phenylalanine, tyrosine, and tryptophan biosynthesis pathway, as well as the phenylalanine metabolism pathway, may be involved in HCC genesis and the TACE treatment process. Consistent with our results, Jee *et al*^17^ found abnormalities in these pathways in patients with HCC relative to healthy volunteers. To date, there have been few reports on differences in metabolites or pathways before and after TACE in patients with HCC. The key amino acids involved in these two pathways are phenylalanine and tyrosine. In this study, phenylalanine and tyrosine were elevated in the plasma of the pre-TACE group compared with the healthy group, consistent with previous reports^18^. In addition, phenylalanine, but not tyrosine, was further increased after TACE. These two amino acids participate in the synthesis of important neurotransmitters and hormones, such as dopamine and thyroid hormone^19^ and abnormal metabolism of tyrosine is associated with HCC occurrence^20^. Phenylalanine, as the precursor of tyrosine, is converted to tyrosine by phenylalanine 4-hydroxylase. Tumor inflammation and immune activation interfere with phenylalanine conversion in ovarian carcinoma by attenuating phenylalanine 4-hydroxylase activity, resulting in increased phenylalanine concentration^21^. Moreover, inflammation and immune activation can increase the plasma phenylalanine/tyrosine ratio^22^. We suspect that this phenomenon also occurs in HCC, leading to elevated plasma phenylalanine and tyrosine compared with healthy controls. The mechanism underlying the increased level of phenylalanine after TACE is not well understood; however, it is plausible that elevated plasma phenylalanine levels may be related to changes in tumor metabolism or immune responses after TACE. Previous studies on the effect of TACE on immune status in HCC showed that Th17 cells and the CD4/CD8 ratio increased, while regulatory T cells significantly decreased, in the periphery after TACE, representing an immune-activated environment^23^,^24^. Thus, this immune-activated environment following TACE may be responsible for the further elevation of phenylalanine compared with those in patients pre-TACE; however, another study also indicated that cytokines associated with Th2 cells were elevated after TACE, leading to an immune suppressive environment^25^. Therefore, the reason for the increase in phenylalanine after TACE in HCC requires further exploration.

ROC curve analysis comparing the healthy and pre-TACE groups showed that palmitoylcarnitine is a potential biomarker for HCC auxiliary diagnosis. We found that palmitoylcarnitine was increased in plasma from patients with HCC compared with the healthy group, consistent with the results of a previous study^26^. Contrary to our findings, Jee *et al* found that palmitoylcarnitine levels were lower in patients with HCC than in healthy controls^17^. Palmitoylcarnitine is a long chain acylcarnitine. The main function of palmitoylcarnitine is as a carrier for transporting long-chain fatty acids into the mitochondria for β-oxidation, which provides energy for cell activities^27^. Palmitoylcarnitine stimulates the early growth of HepG2 cells^28^, which may explain the elevated levels of palmitoylcarnitine in HCC, which represent a marker with potential for application in HCC auxiliary diagnosis.

Finally, ROC curve analysis between the non-tumor response and tumor response groups after TACE showed that PC 36:4|PC 18:2_18:2, which was decreased in the tumor response group, may be a useful marker for evaluating the therapeutic effects of TACE; similar results have not been reported previously. PC 36:4|PC 18:2_18:2 belongs to the class of phosphatidylcholines (PCs), and an association between PCs and HCC has been reported in many studies. Further, PCs are established as involved in lipid metabolism reprogramming^29^,^30^,^31^. A recent study showed that overexpression of lysophosphatidylcholine (LPC) acyltransferase 1 catalyzed the transformation of LPCs to PCs in the Lands PCs biosynthesis cycle, which could enrich PC species and promote cell proliferation in HCC^32^. Moreover, PCs are reported to promote tumorigenesis and metastasis^33^,^34^. Thus, a decline in PCs may predict tumor response after TACE in patients with HCC.

A limitation of our study is the small sample size used, which may impact the generalizability of our findings. Future research efforts with larger and more diverse cohorts are warranted to validate and extend our current findings, enhancing the reliability of the conclusions drawn from this study.

## 5. Conclusion

In this study we aimed to investigate metabolic changes in patients with HCC and to identify potential markers for HCC diagnosis and prognosis assessment after TACE. By analyzing metabolomic profiles, we identified several differential metabolites and related metabolic pathways that may function in HCC development and response to TACE treatment. Our findings suggest that palmitoylcarnitine is a potential marker for HCC auxiliary diagnosis and that PC 36:4|PC 18:2_18:2 could serve as a marker for evaluating the therapeutic effects of TACE; however, our study was limited by a relatively small sample size, and further studies with larger patient cohorts are needed to validate our findings.

## Supplementary Material

Supplementary figures.

## Figures and Tables

**Table 1:** Clinical characteristics of subjects in the HCC and healthy groups

	HCC (n = 20)		Healthy (n = 20)	*p*
	Pre-TACE	Post-TACE		
Male/Female	14/6		13/7	0.736
Age (years)	61 ± 8		57 ± 6	0.229
Pathogenesis (HBV/HCV)	18/2			
AFP (ng/mL)	179.82 ± 237.64	117.56 ± 166.55		0.277
ALT (U/L)	45.84 ± 10.68	52.37 ± 8.69		0.517
AST (U/L)	36.70 ± 3.28	44.35 ± 5.55		0.192
Albumin (g/L)	42.23 ± 2.10	41.03 ± 1.80		0.660
TBIL (μM/L)	19.84 ± 2.06	23.66 ± 3.19		0.189
ALP (U/L)	100.20 ± 8.89	99.11 ± 9.74		0.773
Platelet (10^9^/L)	102.30 ± 10.17	103.4 ± 11.98		0.940

Note: HCC, hepatocellular carcinoma; TACE, transcatheter arterial chemoembolization; HBV, hepatitis B virus; HCV, hepatitis C virus; AFP, alpha-fetoprotein; ALT, alanine transaminase; AST, aspartate transaminase; TBIL, total bilirubin; ALP, alkaline phosphatase.

**Table 2:** Summary of pathways differing between the pre- and post-TACE groups

Pathway	Total^a^	Hits^b^	-LOG(p)	FDR^c^	Impact^d^
Phenylalanine, tyrosine, and tryptophan biosynthesis	4	1	2.47	0.0045	0.50
Phenylalanine metabolism	10	1	2.47	0.0045	0.36
Aminoacyl-tRNA biosynthesis	48	1	2.47	0.0045	0
Fatty acid degradation	39	1	1.65	0.022	0

^a^Total number of compounds in the pathway ^b^Number of plasma metabolites involved in the pathway ^c^False discovery rate (FDR) was analyzed using the “fdrtool” package in the R platform ^d^Pathway impact value calculated by pathway topology analysis

**Table 3:** List of significant metabolites identified by ROC curve analysis

Metabolite	Sensitivity	Specificity	95% CI	Cut off value
PC 36:4|PC 18:2_18:2	66.67%	81.82%	0.561-0.942	≤ 7.348
LPC 22:0	88.89%	72.73%	0.528-0.924	≤ 0.013

Note: CI, confidence interval

## Abbreviations

HCC: Hepatocellular carcinoma
TACE: transcatheter arterial chemoembolization
ROC: receiver operating characteristic
UHPLC-HRMS/MS: liquid chromatography-high resolution mass spectrometry
PCA: principal component analysis
OPLS-DA: orthogonal filtered partial least squares discriminant analysis
HBV: hepatitis B virus
HCV: hepatitis C virus
AFP: alpha-fetoprotein
ALT: alanine transaminase
AST: aspartate transaminase
TBIL: total bilirubin
ALP: alkaline phosphatase
