# Supplementary material for: Plasma metabolomic analysis of human hepatocellular carcinoma before and after transcatheter arterial chemoembolization
Source: Int J Med Sci. 2024 Jan 1;21(2):413–23. doi: 10.7150/ijms.89141 (PMC10758137; doi:10.7150/ijms.89141)
Supplement: Supplementary file 1 — Supplementary figures. [file ijmsv21p0413s1.pdf]

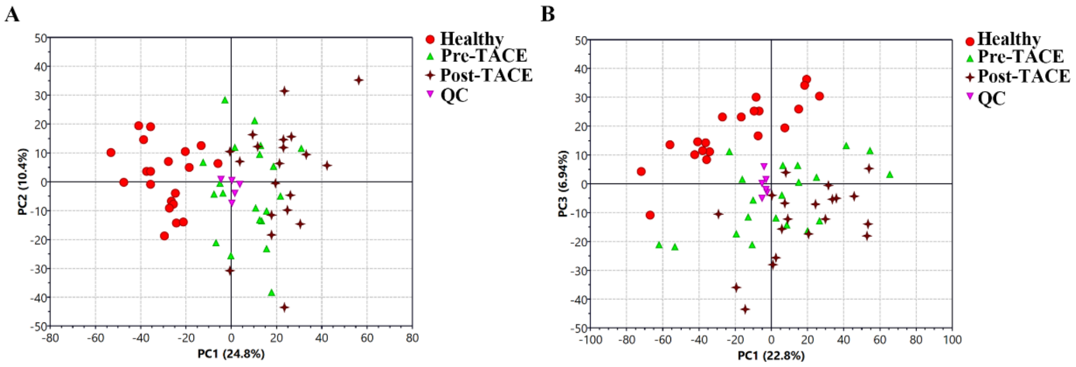

Supplemental Figure 1. The PCA plot for healthy, pre-TACE and post-TACE groups as well as QC. (A) The positive ion mode; (B) The negative ion mode. $R^2X=0.679$ for positive ion mode and $R^2X=0.511$ for negative ion mode. $R^2X > 0.5$ indicates reliable model quality.

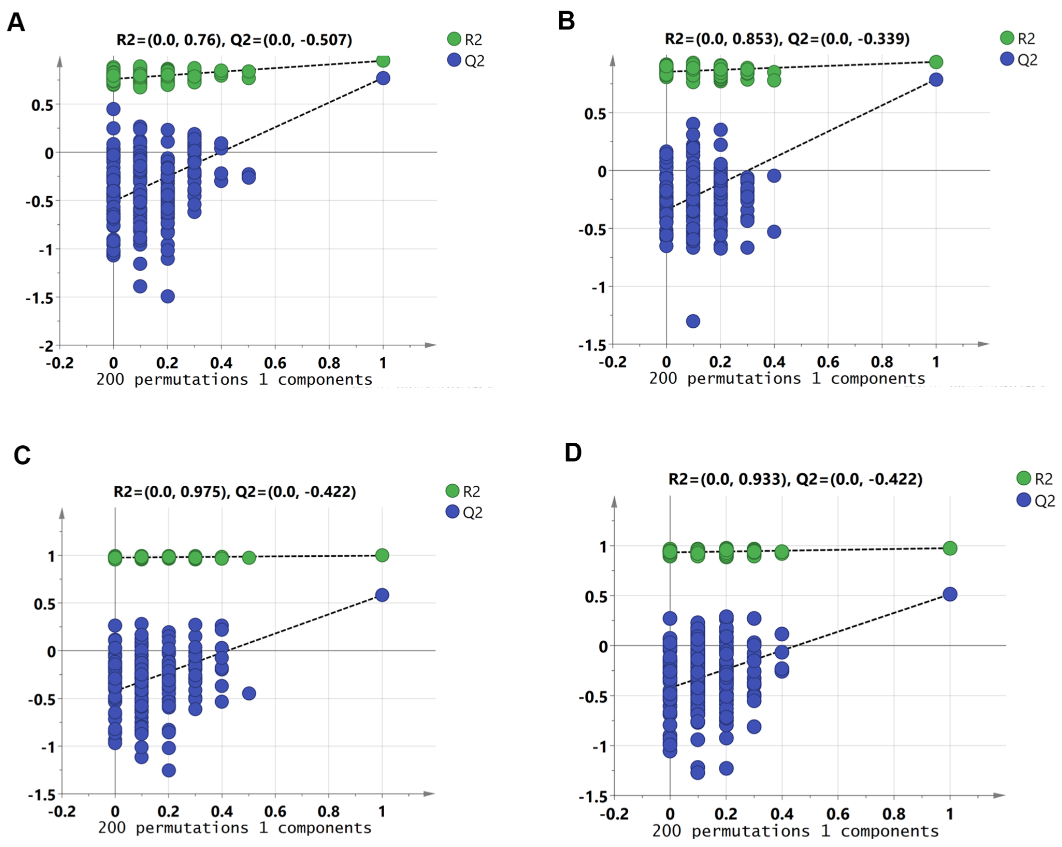

Supplemental Figure 2. The permutation tests for OPLS-DA model. (A, B) The permutation tests for positive ion and negative ion between healthy and pre-TACE groups, respectively. (C, D) The permutation tests for positive ion and negative ion between pre-TACE and post-TACE groups, respectively.

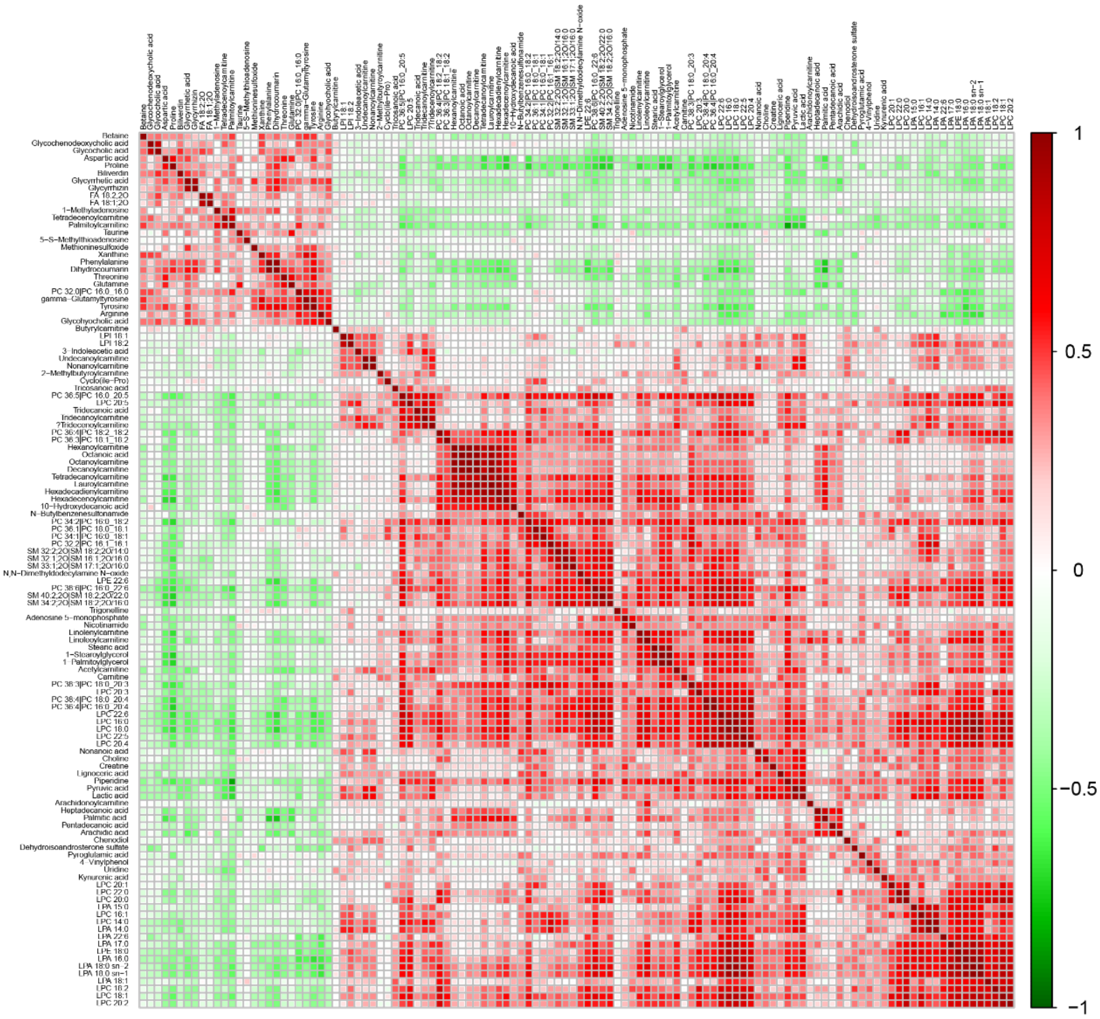

Supplemental Figure 3. Correlation matrix of different metabolites in healthy and pre-TACE groups. The Pearson Correlation was conducted for the quantitative information of these metabolites.

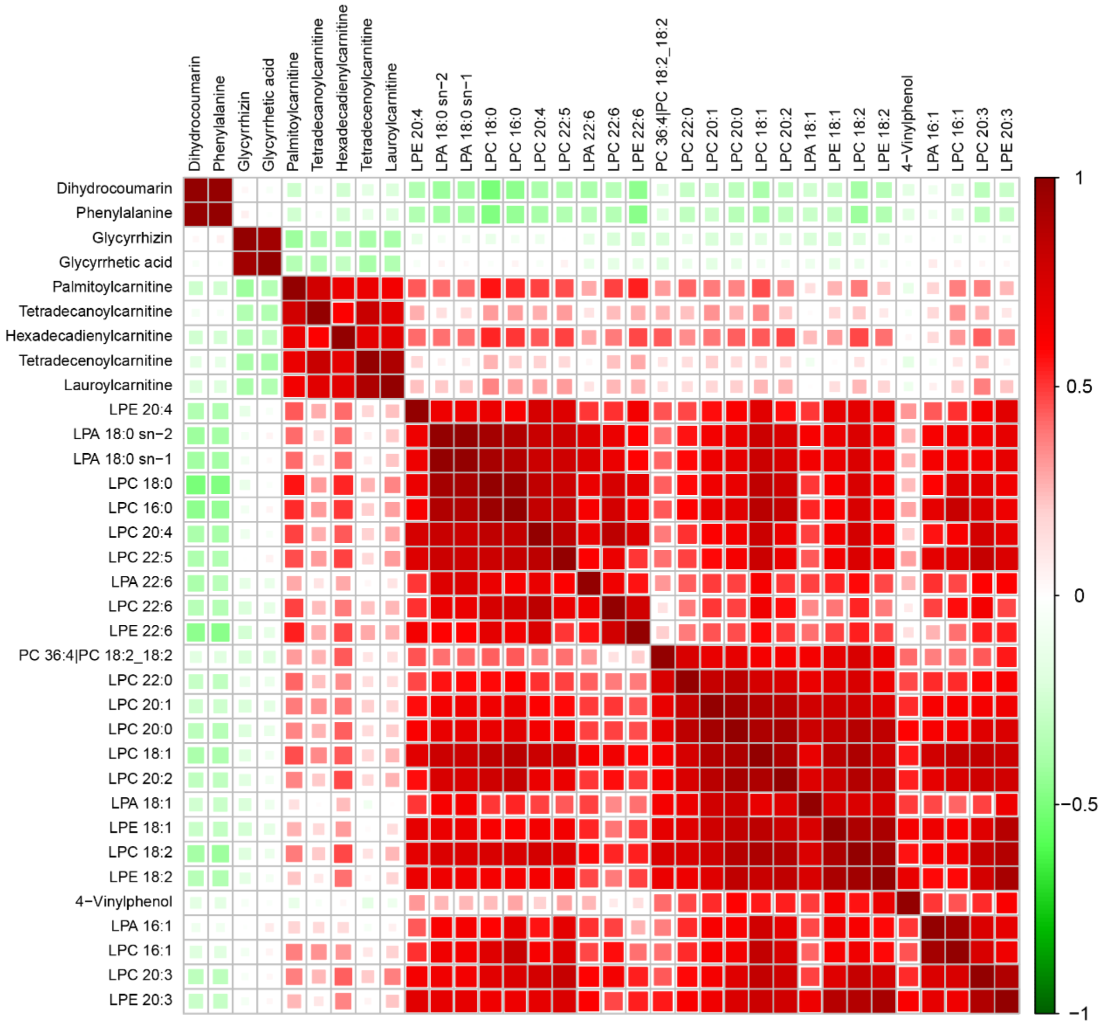

Supplemental Figure 4. Correlation matrix of different metabolites in pre-TACE and pro-TACE groups. The Pearson Correlation was conducted for the quantitative information of these metabolites.

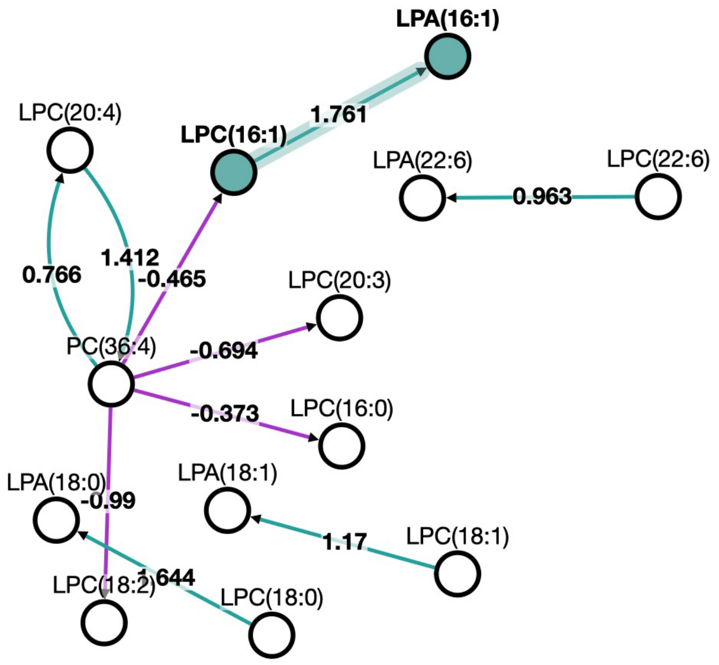

Supplemental Figure 5. Lipid network graphs exported from BioPAN from the 34 differential metabolites between pre-TACE and post-TACR groups. Reactions with a positive Z score have green arrows while negative Z scores are colored purple
